# METASTATIC CROHN'S DISEASE OF EXTERNAL GENITALIA

**DOI:** 10.4103/0019-5154.43207

**Published:** 2008

**Authors:** Charles Panackel, Joseph John, Devadas Krishnadas, Kattoor R Vinayakumar

**Affiliations:** *From the Department of Medical Gastroenterology, Medical College Trivandrum, Kerala, India - 695 004*

**Keywords:** *Extraintestinal manifestation of IBD*, *metastatic Crohn's disease*, *noncaseating granuloma*

## Abstract

Metastatic Crohn's disease is an uncommon extraintestinal manifestation of Crohn's disease. Its hallmark features include the presence of cutaneous noncaseating granulomas that are noncontiguous with the gastrointestinal tract or fistula. We report a rare case of metastatic Crohn's disease involving the external genitalia in a 14-year-old girl. Diagnosis was based on skin biopsy. Patient had complete recovery on treatment with oral and topical steroids along with azathioprine.

## Introduction

Metastatic Crohn's disease is an uncommon extraintestinal manifestation of Crohn's disease. Its hallmark features include the presence of cutaneous noncaseating granulomas that are noncontiguous with the gastrointestinal tract or fistula. We report a rare case of metastatic Crohn's disease involving the external genitalia in a girl with Crohn's disease and discuss the various manifestations and treatment options.

## Case History

**Fig. 1:**
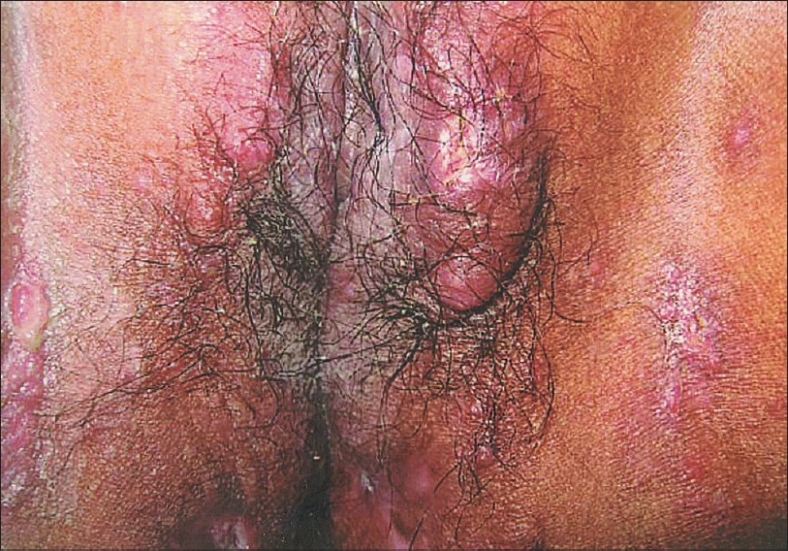
Multiple papules, nodules and ulcers on the external genitalia and surrounding skin

**Fig. 2:**
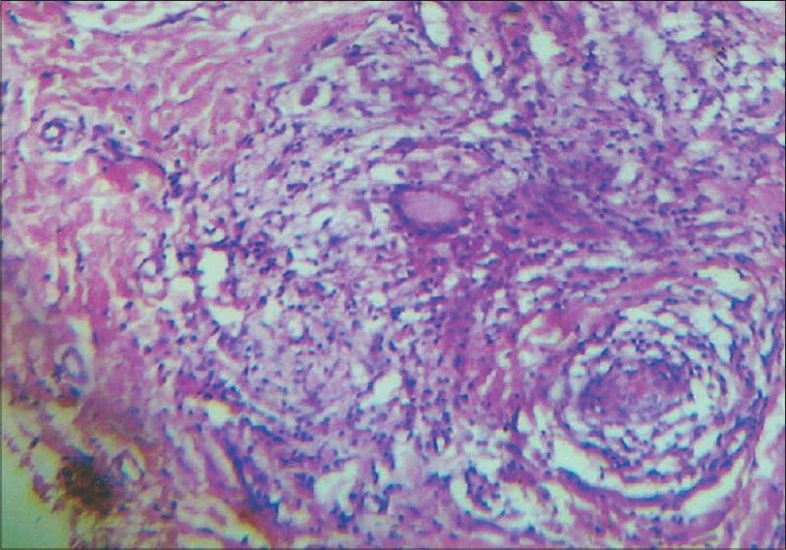
Skin biopsy showing noncaseating granuloma (H&E, ×100)

**Fig. 3:**
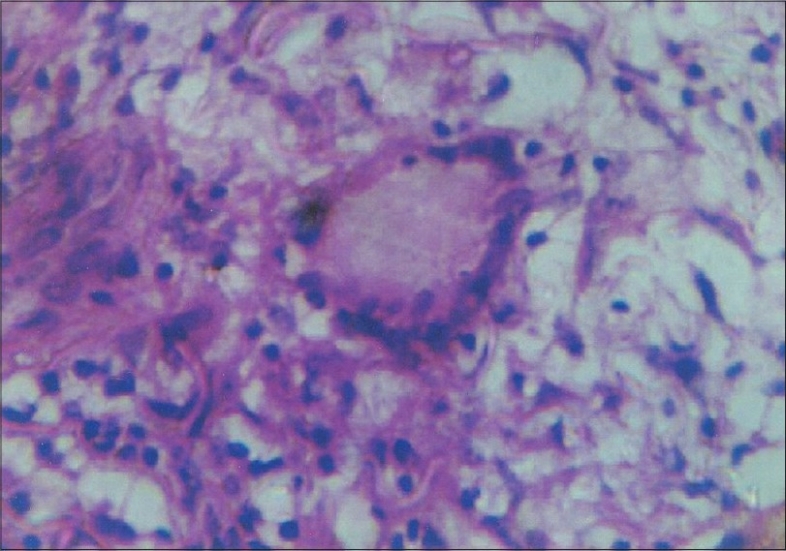
Skin biosy showing multinucleate giant cell and epitheloid cells (H&E, ×400)

A 14-year-old girl presented with history of pain, itching, swelling and ulceration of external genitalia of three weeks duration. Patient was diagnosed to have pulmonary tuberculosis in 2000 for which she took Antitubercular treatment for six months. In 2002, she presented with chronic diarrhea and was diagnosed as intestinal tuberculosis. She was re-treated with antitubercular drugs with no relief of symptoms. One year later she presented with perianal abscess and erythema nodosum when a repeat colonoscopy showed apthoid ulcers, cobble stone appearance, fissuring ulcers and pseudopolyps in ascending, transverse, descending and sigmoid colon. Ileum, ileocaecal valve and Caecum were normal. A barium meal follow through was normal. Colonic biopsy showed evidence of Crohn's disease and a diagnosis of extensive Crohn's disease with moderate activity was made (CDAI-260). She was initiated on treatment with antibiotics and mesalamine followed by steroids. In 2005, she presented with recurrent relapse of symptoms while on steroids. She was diagnosed to have steroid refractory Crohn's and was started on azathioprine(100mg/day). She was in remission (CDAI-146) when she developed pain, itching of external genitalia followed by papular eruptions and ulceration (Fig. 1). There was no fever, constitutional symptoms or vaginal discharge. She had no aggravation of her bowel symptoms. A skin biopsy was done which was inconclusive and clinical diagnosis of herpes simplex infection was made. Patient was started on acyclovir with no relief of symptoms. A repeat skin biopsy was done which showed noncaseating epitheloid granulomas, multinucleated giant cells, and perinuclear cuffing with lymphocytes (Figs. 2 and 3). Gram, Zeil Nielson and period acid Schiff stain were negative. A diagnosis of metastatic Crohn's disease was made and oral prednisolone 40 mg and topical steroids were added to azathioprine. Steroids were continued for four weeks and tapered. She had complete relief of symptoms. All throughout her present illness her Crohn's disease remained in remission.

## Discussion

Cutaneous manifestations occur in 22-44% of patients with Crohn's disease.^1^ Three distinct patterns of cutaneous involvement are seen in Crohn's disease. The most common is the peristomal or perianal disease where the gastrointestinal tract disease encroaches on the adjacent skin. Second pattern includes a variety of conditions associated with Crohn's disease like erythema nodosum, pyoderma gangrenosum, neutrophilic dermatosis, steven Johnson syndrome, erythema multiforme, acrodermatitis enteropathica, and epidermolysis bullosa acquista.^2^ The third type is the metastatic Crohn's disease. The disease was first described by parks *et al*, in 1965.^3^ It was Mountain who coined the term metastatic Crohn's disease in 1970.^4^

Metastatic Crohn's disease is characterized by noncaseating granulomatous involvement of skin noncontiguous from the gastrointestinal tract in a patient with Crohn's disease.^5^ Metastatic Crohn's disease of the external genitalia is exceedingly rare, with few documented cases in the literature. Metastatic Crohn's disease can have a varied presentation. It can present as cutaneous ulcerations, plaques, papules and nodules.^6^ Lesions usually have a predilection for skin folds, infra-mammary area and the limbs. Biopsies from these lesions show multinucleated giant cells, noncaseating granulomas, perivascular lymphocytes, monocytes and necrobiosis.^5^,^7^–^9^ Metastatic Crohn's disease appears to be more common in patients with colonic involvement. There is no relation between presence of metastatic Crohn's and activity of intestinal disease. Metastatic Crohn's disease can precede or occur along with gastrointestinal disease. Our case had papules, nodules and ulcers of external genitalia. She had Crohn's colitis which was in remission.

Other conditions that can be mistaken for metastatic Crohn's include syphilis, tuberculoid leprosy, sarcoidosis, tuberculosis, fungal infections, foreign body granulomas and erysipelas. A definite diagnosis requires a thorough evaluation including biopsy and cultures.

There are no definite guidelines for treatment of metastatic Crohn's. Drugs tried include topical agents, oral steroids, aminosalicylates, immunosuppressive agents, oral antibiotics, hyperbaric oxygen^10^ and surgery.^11^ Infliximab an antitumor necrosis factor-a chimeric monoclonal antibody is used for the treatment of Crohn's disease. Till date there are six case reports where Infliximab was used to successfully treat metastatic Crohn's disease.^12^–^17^ Infliximab has been successfully used to treat other cutaneous manifestations of Crohn's disease like pyoderma gangrenosum and hidradenitis suppurative. Our patient was continued on azathioprine and both topical and oral steroids were added. Oral steroids were continued in a dose of 1 mg/kg for four weeks and taper gradually till complete healing occured.

## Conclusion

Metastatic Crohn's disease is a disfiguring illness and often refractory to treatment. A thorough evaluation including biopsies is required to make a definite diagnosis. Ours is a case of metastatic Crohn's disease involving external genitalia and it responded to oral and topical steroids along with azathioprine.
